# How I Do It: A Novel Case Series of Office‐Based Laser Complete Excision of Vocal Fold Cysts

**DOI:** 10.1002/lary.70627

**Published:** 2026-05-15

**Authors:** Aurora Ka Yue Tam, Timothy Shun Man Chu, Yifeng Wei, Zikai Wang, Thomas Law, Jason Ying Kuen Chan

**Affiliations:** ^1^ Department of Otorhinolaryngology, Head and Neck Surgery The Chinese University of Hong Kong Hong Kong China; ^2^ The Institute of Human Communicative Research The Chinese University of Hong Kong Hong Kong China

**Keywords:** complete vocal fold cyst excision, office‐based laser, vocal cord cyst, vocal fold cyst

## Abstract

Office‐based complete excision of vocal fold cysts using Trublue laser via transnasal flexible endoscopy is a technically feasible alternative to conventional marsupialization, enabling en bloc removal under local anesthesia with excellent procedural tolerability.

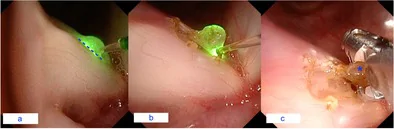

## 1 Introduction

Office‐based awake transnasal laser‐assisted surgery (TNLS) has gained increasing recognition for treating benign laryngeal lesions, offering distinct advantages including avoidance of general anesthetic risks, minimized healthcare‐related costs, and shortened hospital length of stay. Our center's recent randomized controlled trial demonstrated that TNLS achieves functional outcomes comparable to microlaryngeal surgery (MLS) performed under general anesthesia [1, 2].

However, vocal fold cysts represent a distinct therapeutic challenge within the spectrum of TNLS applications. While TNLS has been successfully employed for various benign laryngeal lesions including polyps, nodules, and Reineke's edema, the majority of current local anesthetic (LA) approaches for vocal fold cysts utilize marsupialization (wide‐opening technique) rather than complete excision [3].

The preference for marsupialization stems from technical difficulties in achieving precise cyst dissection using laser under LA, while preserving as much superficial lamina propria and preventing vocal ligament injury, scarring, and subsequent voice impairment. Historically, complete excision using the microflap technique under general anesthesia has been associated with a reported recurrence rate of 2.2%–8.7%, while recurrence rates following LA marsupialization range from 4.7%–18.4% [3, 4]. Notably, our center's randomized controlled study revealed a higher overall vocal fold cyst recurrence rate of 13.8% in the TNLS group compared to historical cohorts, potentially attributable to the laser ablation and marsupialization approach rather than complete cyst removal [2]. This finding highlights the need for technical refinement to enable complete cyst excision under TNLS; here we describe our technique and present clinical and voice outcomes from a pilot feasibility series.

## 2 Methods

### 2.1 Ethics

The study was approved by the Joint Chinese University of Hong Kong–New Territories East Cluster Clinical Research Ethics Committee (CREC 2023.644 T). Informed consent was obtained.

### 2.2 Subjects

Patients with vocal fold cysts who underwent LA Trublue laser excision between November 2024 and June 2025 were prospectively recruited in a tertiary referral hospital in Hong Kong.

### 2.3 Surgical Procedure

The surgical technique can be viewed in the online Video 1 and is described here. Transnasal endoscope approach was adopted using Olympus ENF‐VT3 flexible endoscope. Local anesthesia was achieved using 5% Lidocaine/0.5% Phenylephrine (Co‐phenylcaine) nasal spray and 2% Xylocaine laryngeal drip via endoscopic catheter. During application of laryngeal Xylocaine, the patient was asked to say ‘e’ to perform laryngeal gargle to facilitate distribution of the local anesthesia (Figure 1). The adequacy of anesthesia was confirmed with absence of gag reflex upon gentle palpation of supraglottis with the tip of the endoscope. For procedures exceeding 25 min, additional 2% Xylocaine laryngeal drip was applied as needed to maintain comfort.

**VIDEO 1:** Office‐based complete excision of vocal fold cyst. The video demonstrates office‐based complete excision of a vocal fold cyst using a 445 nm Trublue laser via awake transnasal flexible endoscopy, with local anesthesia application, lateral mucosal incision, subepithelial dissection with the laser fiber, and intact cyst removal with bronchoscopic forceps. Video content can be viewed at https://onlinelibrary.wiley.com/doi/10.1002/lary.70627.

**FIGURE 1:**
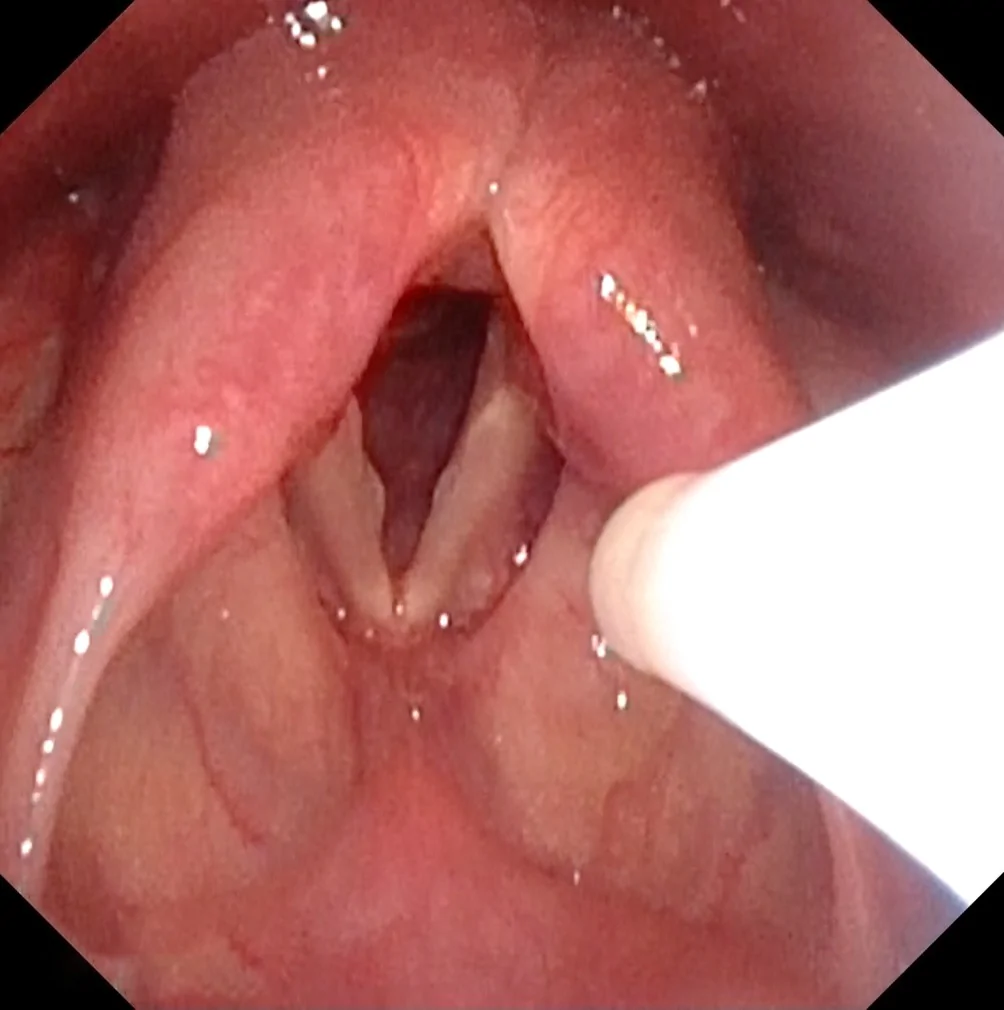
Application of local anesthesia with 2% xylocaine with laryngeal dripping using endoscopic catheter.

A thin 300 μm Trublue laser (445 nm) fiber was introduced via flexible bronchoscope channel, pulsed mode 50 ms on 200 ms off, power setting at 4 W. A lateral mucosal incision was made with the laser, then the cyst was gently dissected away from superficial lamina propria using the tip of the laser fiber as a blunt dissector, while applying the laser as needed to function as the ‘knife’ when encountering adhesive tissues. (Figure 2b). Judicious use of laser during the dissection is prudent in avoiding inadvertent cyst rupture and over‐removal of the superficial lamina propria. Once the cyst is adequately mobilized from the superficial lamina propria, the vocal fold cyst is removed intact with bronchoscopic forceps and can be sent for pathology (Figure 2c). For intracordal cyst, after dissection the medial epithelium can be preserved (Figure 3), while for mucous retention cyst it is usually removed along with the cyst. Hemostasis using laser as needed. Post‐operatively, the patients were kept nil‐by‐mouth for 2 h and discharged on the same day of surgery. Patients were advised to have voice rest for 3 days and were prescribed with oral pantoprazole 40 mg daily for 4 weeks.

**FIGURE 2:**
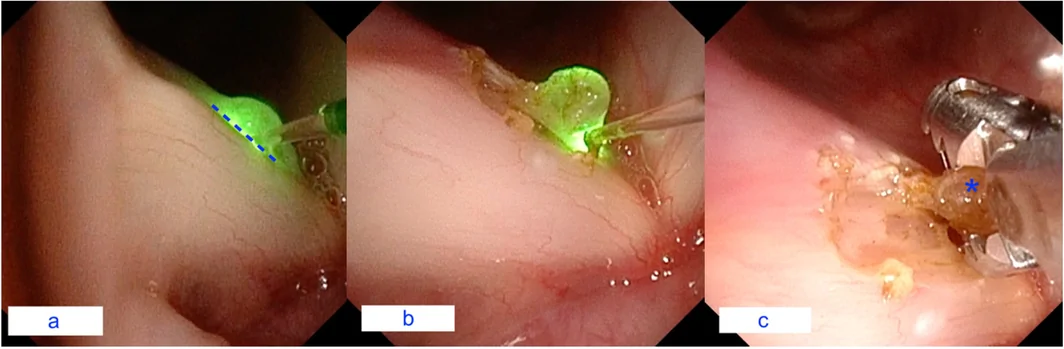
(a) Lateral mucosal incision made with Trublue laser at superior surface of vocal fold. (b) Vocal fold cyst carefully dissected away from using the tip of laser fiber, preserving superficial lamina propria, only applying laser as needed. (c) Vocal fold cyst (Asterisk) removed with bronchoscopic forceps once adequately mobilized.

**FIGURE 3:**
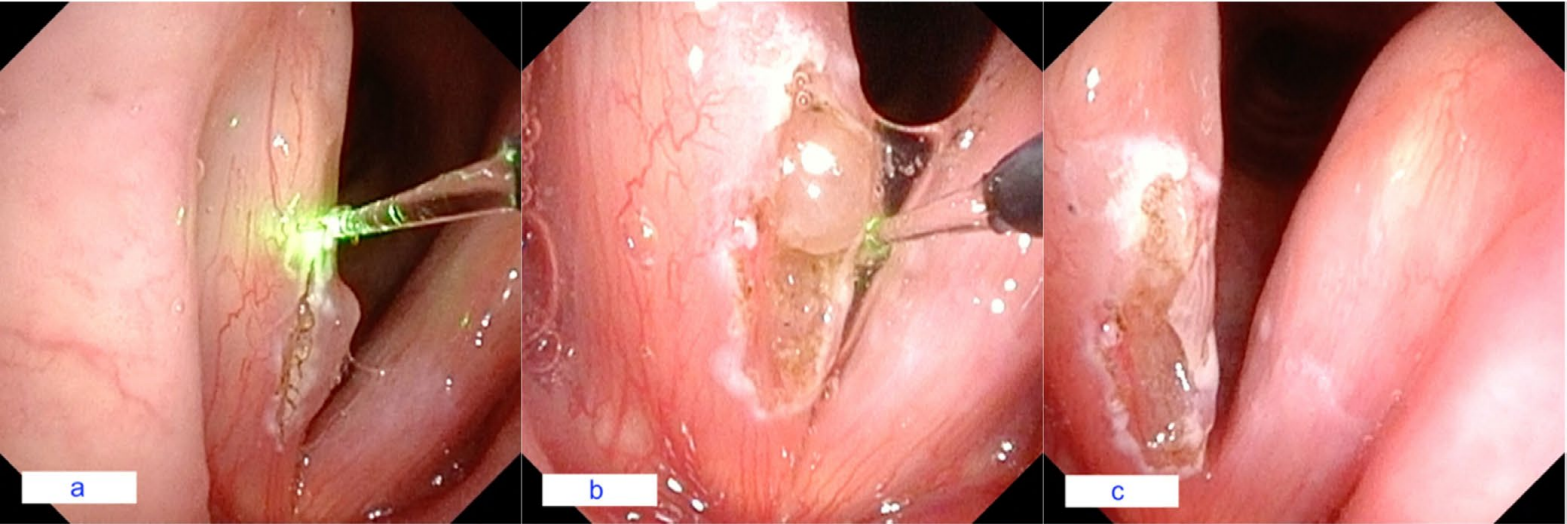
Case showing preservation of medial phonatory mucosal edge post‐excision. (a) Lateral mucosal incision made with Trublue laser at superior surface of vocal fold. (b) Vocal fold cyst carefully dissected from both superficial lamina propria and the medial phonatory edge mucosa. (c) Post‐removal of vocal fold cyst with preserved medial phonatory edge mucosa.

### 2.4 Outcome Measurements

Clinical outcomes include recurrence, operative time, and procedural tolerance measured by the Visual Analogue Scale (VAS).

Preoperative and postoperative voice parameters including Voice Handicap Index‐30 (VHI‐30), Maximum phonation time (MPT), stroboscopy, Cantonese Perceptual Evaluation of Voice (CanPEV), Shimmer and Jitter were analyzed. MPT was measured by instructing patients to sustain the vowel “a” at conversational speech volume for as long as possible, then average of the three attempts were calculated and selected. Acoustic analysis evaluated Jitter and Shimmer using Praat version 6.4.47. Stroboscopy analysis including vocal fold edge, mucosal wave, amplitude and glottic closure pattern.

## 3 Results

### 3.1 Patient Characteristics & Clinical Outcomes

The case series included 5 patients (M:F = 2:3), mean age 59.2 (55–67 yo). One had epidermoid cyst, four had mucus retention cysts. The mean operative time was 32.8 min (20–50 min), with a low level of discomfort with mean pain score of VAS 1.2/10 (0–2) reported. All patients were discharged on the same day of procedure. There were no cyst recurrences observed at a mean follow‐up period of 16.8 weeks (12–24 weeks).

### 3.2 Voice Outcomes

Significant improvements were observed in VHI (34.2 ± 19.4 vs. 14.8 ± 13.1, p < 0.05*), MPT (9.0 ± 7.2 vs. 12.8 ± 8.7 s, p < 0.05*), Shimmer (0.48 ± 0.34 vs. 0.17 ± 0.14 dB, p < 0.05*), overall (6.2 ± 1.3 vs. 4.4 ± 1.14, p < 0.05*) and roughness (5.2 ± 1.3 vs. 3.8 ± 1.1, p < 0.05*) of CanPEV score. No significant improvement was noted in Jitter (0.76 ± 0.63 vs. 0.21 ± 0.05, *p > 0.05*) (Table 1). In terms of stroboscopic ratings, all patients had increased amplitudes of their vocal fold movements and improved mucosal wave movements. Moreover, all patients achieved complete glottic closure post‐op.

**TABLE 1:** Voice outcomes of the five patients with office‐based laser excision of vocal fold cysts performed.

	Pre‐op mean	Post‐op mean on latest FU	Mean change	*p*
VHI‐30 score	34.2	14.8	−19.4	p = 0.043*
MPT (seconds)	9	12.8	+3.8	p = 0.043*
Shimmer (dB)	0.48	0.17	−0.31	p = 0.043*
Jitter (dB)	0.76	0.21	−0.55	*p = 0.08*
CanPEV score: Total roughness	6.2 5.2	4.4 3.8	−1.8 −1.4	p = 0.041* p = 0.038*

*Note:* * *p* < 0.05 is considered as ‘statistically significant’.

## 4 Discussion

Current office‐based approaches for vocal fold cysts predominantly employ marsupialization, which by design leaves residual lateral cyst wall in situ. This technical limitation is reflected in a higher recurrence rate (4.7%–18.4%) compared with complete excision using the microflap technique under general anesthesia (2.2%–8.7%) [3, 4]. Motohashi et al. reported a 4.7% recurrence rate following endoscopic marsupialization under topical anesthesia, with all recurrences requiring reoperation [4]. These findings align with our prior randomized controlled trial which demonstrates a 13.8% recurrence rate in the office‐based arm, where lesions were ablated and marsupialized rather than completely excised.

Our novel technique fundamentally differs by enabling complete cyst excision through precise microdissection and judicious use of laser, under a clear visualization with flexible endoscope. For intracordal cyst, the approach is analogous to microflap surgery performed under general anesthesia, in which with lateral mucosal incision at the superior surface of the vocal fold, the vocal fold cyst is carefully dissected away from superficial lamina propria while preserving the medial phonatory segment of the vocal fold (Figure 3). This alternative approach allows a shift from ablative marsupialization to en bloc resection while maintaining the benefits of an awake, office‐based procedure. The technical key lies in judicious use of the 445 nm blue laser fiber as both a cutting instrument and blunt dissector, facilitating subepithelial plane development while preserving maximal superficial lamina propria.

Our novel approach confers several distinct advantages. Foremost, complete vocal fold cyst excision may reduce the risk of residual disease compared with marsupialization; in this small pilot series we observed no early recurrence over a mean follow‐up of 16.8 weeks, but long‐term recurrence rates cannot be determined from these data. Secondly, our cohort demonstrates significant voice parameter improvement across VHI‐30, MPT, shimmer, and CanPEV scores, while preserving the mucosal wave and restoring a complete glottic closure in all patients. These functional gains mirror those reported in marsupialization series, while offering complete histologic removal. Thirdly, the office‐based approach provides an alternative option for patients unsuitable for or refusing general anesthesia, while keeping a short operative time and good procedural tolerability. The technique may also particularly benefit patients with epidermoid cysts or those requiring pathology confirmation, as en bloc excision provides intact specimens for histologic analysis, unlike fragmented marsupialization specimens.

Of note, our cohort has a small sample size, lacks long‐term follow‐up and concurrent comparator group, which fail to establish long‐term recurrence rates or demonstrate superiority over established marsupialization method. As such, this pilot cohort's findings are hypothesis generating, and future prospective studies with larger cohorts should encompass comparison against conventional marsupialization with longer‐term recurrence surveillance. The technique also demands a good laser knowledge and precise laser manipulation to avoid cyst rupture or excessive superficial lamina propria removal that risks postoperative fibrosis and scarring—thus necessitating a steeper learning curve. Nonetheless, with good anesthetizing techniques and practice, the skillset should be replicable.

## 5 Conclusion

This pilot series demonstrates that office‐based complete laser excision of vocal fold cysts is feasible, effective, and well‐tolerated, achieving complete histologic removal with no short‐term recurrence and good voice outcomes in our cohort. This represents a meaningful technological advancement, particularly for patients who are medically unsuitable for or who decline general anesthesia.

## Funding

The authors have nothing to report.

## Conflicts of Interest

The authors declare no conflicts of interest.

## Data Availability

Research data are not shared.
